# Influence of Algal Incorporation on Sensory and Physicochemical Attributes of Caseless Sausage—Ćevap (CSC)

**DOI:** 10.3390/foods13244037

**Published:** 2024-12-13

**Authors:** Caba Siladji, Vesna Djordjevic, Branka Borovic, Volker Heinz, Nino Terjung, Nenad Katanic, Igor Tomasevic

**Affiliations:** 1 Institute of Meat Hygiene and Technology, Kaćanskog 13, 11000 Belgrade, Serbia; vesna.djordjevic@inmes.rs (V.D.); branka.borovic@inmes.rs (B.B.); 2 DIL German Institute of Food Technologies, Prof.-v.-Klitzing-Str. 7, 49610 Quakenbrueck, Germany; v.heinz@dil-ev.de (V.H.); n.terjung@dil-tec.de (N.T.); 3 Ministry of Agriculture, Forestry and Water Management, Nemanjina 22-26, 11000 Belgrade, Serbia; nenad.katanic@minpolj.gov.rs; 4 Faculty of Agriculture, University of Belgrade, Nemanjina 6, 11080 Belgrade, Serbia

**Keywords:** *Chlorella*, sea spaghetti, wakame, minced meat product, sausages

## Abstract

This study explores the influence of algal incorporation on the sensory and physicochemical attributes of caseless sausage—ćevap (CSC). Various algae, including *Chlorella vulgaris*, *Himanthalia elongata* (sea spaghetti), and *Undaria pinnatifida* (wakame), were added at different concentrations to standard CSC formulations. Proximate analysis revealed that the addition of algae did not significantly change crude protein and fat content (*p* > 0.05). Furthermore, sea spaghetti and wakame resulted in lower moisture levels and decreased cooking loss, while all algae varieties raised the salt content (*p* < 0.05) due to their inherent sodium levels. Texture analysis demonstrated that the inclusion of sea spaghetti and *Chlorella* led to reductions in hardness and chewiness, while wakame resulted in a firmer texture, highlighting its substantial impact on textural attributes. Color measurements indicated that wakame significantly affected the color profile (*p* < 0.05), resulting in decreased lightness and increased darkness in the sausage, surpassing the effects of other algae. Sensory evaluations showed that formulations containing wakame received the lowest scores for color, smell, taste and overall acceptability—up to 1.5 points lower compared to the control samples. On the other hand, some formulations with other algae showed promising small deviations from the control. Overall, this research supports the viability of using algae as functional ingredients in meat products, emphasizing the importance of selecting the appropriate type and concentration of algae to optimize both physicochemical parameters and sensory qualities in caseless sausages.

## 1. Introduction

The food industry and consumers are increasingly focusing on products that promote health and well-being. These foods, commonly referred to as functional foods, offer health advantages that extend beyond basic nutrition [1]. This trend is also evident in the meat industry, where functional meat products are gaining popularity globally [2]. As meat consumption continues to rise, particularly in developing countries [3,4], there is a growing demand for meat products that not only satisfy dietary preferences but also contribute to better health outcomes. Functional meat products, enriched with ingredients such as omega-3 fatty acids, antioxidants, and probiotics, are becoming more prevalent in the market. These products meet customers’ demands for healthier options and answer concerns about health issues related to diet, aligning with the broader trend toward functional foods [5].

Incorporating algae into food products represents a promising development due to their high nutritional value and potential health benefits. They serve as an excellent source of proteins, lipids, carbohydrates, vitamins, and macro- and microelements [6,7,8], while their metabolites exhibit various beneficial health effects, including antioxidant, anti-inflammatory, anti-cancer, and antibacterial activities [9,10,11]. Additionally, the sustainable cultivation of algae aligns with environmental concerns, as they require fewer resources than traditional crops and can help reduce the carbon footprint of food production [12].

Microalgae, such as *Chlorella* and *Spirulina*, have a high protein content, reaching up to 70% on a dry weight basis. In comparison, the protein content in different macroalgae species varies, ranging from 10% in *Fucus vesiculosus* to 47% in *Porphyra tenera* [13]. The most commonly used algae-derived ingredients include carrageenan, agar, and alginates. These polysaccharides are widely utilized in various food products for their thickening, gelling, stabilizing, and packaging properties [14].

A recent review conducted by Siladji et al. [15] highlighted the impact of incorporating algae into meat products. The findings indicate that certain types of algae when added in higher concentrations, can enhance the nutritional profile of these products. Additionally, other studies have demonstrated that while total protein and fat content may show only slight improvements, the inclusion of algae significantly enhances the amino acid and fatty acid profiles of frankfurters [16,17], meat emulsion systems [18], and cooked turkey breasts [19].

Grilled minced meat is a popular food choice worldwide, celebrated for its flavor, versatility, and cultural significance. In Western countries, it is commonly found in the form of burgers and sausages, while in Southeastern Europe, it is popular as kebabs, koftas, pljeskavicas (pronounced/pʎ$\hat{ɛ}$skaʋitsa/), and ćevaps (pronounced/t͡ɕěʋaːp/). These types of meat products are highly valued for their sensory characteristics, high-quality proteins, and other essential nutrients. Fatty tissue and salt are important components in the formulation of minced meat products due to their significant impact on sensory attributes. Salt affects taste, shelf-life, safety, and texture, while also promoting protein solubilization and extraction, enhancing water-holding capacity [20]. At the same time, meat quality, the proportion of different meat types in the product, and different spices are also crucial.

Given the high consumption of these fast-food products in the Balkans, there is a need to improve the nutritional parameters of grilled minced meat products. In Serbia, one of the most popular forms of these foods is caseless sausages—ćevaps (CSC), which are formed into a cylindrical shape (approximately Ø 2 cm, 6–8 cm in length) [21]. Consumers are familiar with this product due to its recognizable color, texture, and taste, perceiving it as a national specialty with a long tradition.

This article hypothesizes that it is possible to enhance CSCs with different types of algae, while preserving color, texture, and sensory parameters as much as possible, thereby creating a nutritionally improved meat product. In this context, the main goal of this research was to define the type and concentration of algae that can be incorporated into the CSC formulation to improve the product while minimizing any changes to its physicochemical and sensory characteristics compared to the original formulation.

## 2. Materials and Methods

### 2.1. Ingredients

Fresh post-rigor pork and beef meat with fat were obtained from Landschlachterei G.H. Diekmann (Essen Oldenburg, Germany). White *Chlorella vulgaris* powder was purchased from Aliga microalgae (Hjørring, Denmark), while sea spaghetti (*Himanthalia elongata*) and wakame (*Undaria pinnatifida*) powders were purchased from Alganex (Berlin, Germany).

### 2.2. Meat Preparation

The CSCs used in this study were prepared at the German Institute of Food Technologies (DIL e.V., Quakenbrück, Germany) following an industrial processing protocol. The entire study occurred over three consecutive days, with each group being prepared in triplicate. On day seven, different CSC formulations (Table 1) were prepared following a standard industrial recipe as follows: 89% meat mixture with fat (49% beef, 40% pork) and 11.0% ice water, while 1.4% salt, 0.6% dextrose, and algae (1.5% and 3%) were added “on top”. The pork and beef meats were standardized according to the GEHA meat classification system [22], with pork standardized to S III (with 12% fat) and beef to R II (with 8% fat). The meat was ground through a 7.8 mm sieve, salted with NaCl, covered with foil, and stored overnight at 4 °C. Afterward, dextrose, salt, and algae were added to all formulations (except the control group, which contained no algae), and mixed using a bowl chopper (5000 Express, 30 l, KILIA GmbH, Birmingham, UK). The mixture was then shaped into cylindrical forms, approximately 2 cm in diameter and 8 cm in length, using a vacuum filler (VF 608 plus, Albert Handtmann Maschinenfabrik, Biberach der Riss, Germany). After being shaped, the specimens were allowed to rest for four hours at 4 °C, and then they were cooked on an electric grill (GGM Gastro International, Gronau, Germany) until the internal temperature reached 75 °C.

### 2.3. Measurements

Crude proteins of CSC were analyzed by the Kjeldahl method, previously performed by Schiel, et al. [23]. Results were converted with a nitrogen-to-protein conversion factor of 6.25. Contents were calculated as the crude protein per 100 g sample. Fat content was determined by the Caviezel method, previously performed by Baune, et al. [24]. Both crude protein and fat content are described in §64 LFGB L 06.00-6 2014-08 [25]. Moisture content was examined with the sea–sand method as performed by Witte et al. [26], described in §64 LFGB L 06.00-3 2004-07 [25]. Moisture content was calculated per 100 g and examined in a technical triple determination for each replicate, resulting in *n* = 9. The pH of each CSC was measured directly using Testo 205 (Testo AG, Lenzkirch, Germany). Before the measurements, the pH meter was calibrated at room temperature (20 °C) with buffers at pH 4.00 and 7.00. The measurement was taken three times on days 0, 1, 3, and 7 for each batch, changing the location of electrode insertion, resulting in *n* = 9. The water activity (a_w_-value) was measured at 20 °C using an a_w_ meter Aqualab 4TEV (METER Group, Pullman, WA, USA). The measurements were taken on the day of the production in duplicates, resulting in *n* = 6. Cooking loss was determined by calculating the weight differences before and after cooking in triplicates.

Texture profile analysis (TPA) was completed using TA.XT2 Texture Analyser (Stable Micro System, Godalming, UK). Ten grilled CSCs from each sample were tested in two replicates: sample size cylinder height 25 mm, diameter 35 mm. For the TPA tests, instrumental parameters were pre-test speed—180 mm/min, test speed—60 mm/min, post-test speed—180 mm/min, target mode—distance (50%), and probe selection—P/100 (100 mm compression platen), with a cylindrical sample shape cylindrical and 10 s between cycles. Texture Expert Exceed was used to calculate the values of hardness (N), springiness (mm), cohesiveness, and chewiness (N × mm). The dimensions of the samples were measured using a digital caliper. Preparation of the samples was performed using thin-bladed sharp knives to minimize damage [27].

The color of the samples (both surface and longitudinal section) was measured with a Computer Vision System (CVS), as described in Tomasevic et al. [28], after approximately 20 min of blooming time at room temperature. The total color difference (ΔE) of each batch with alga (s) was calculated regarding the control CSC (c) via Equation (1). A total of 10 measures in three replicates from each sample were used for further analysis.

(1) $$\Delta E=\sqrt{{\left(a_{s}^{*}-a_{c}^{*}\right)}^{2}+{\left(b_{s}^{*}-b_{c}^{*}\right)}^{2}+{\left(L_{s}^{*}-L_{c}^{*}\right)}^{2}}$$

The sensory analysis was completed using Smart Sensory Box software (Smart Sensory Solutions S.r.l., Sassari, Italy). In two sessions performed under identical conditions, fifteen panelists including staff and students at the German Institute of Food Technologies evaluated the CSC acceptability using a seven-point hedonic scale (1 = dislike extremely, 7 = like extremely). To prepare the assessors for the sensory analysis, four two-hour training sessions were conducted over two weeks, following the recommendation of Djekic et al. [29]. The panelists were chosen based on their frequency of meat and meat product consumption (at least twice a week). The attributes evaluated by the panel were color, smell, taste, texture, juiciness, and overall acceptability. During the evaluation, evaluators randomly received a CSC of each sample labeled with a randomized three-digit number in broad daylight. Before evaluation, panelists were informed of the composition of the CSC (pork and beef meat, fat, and algae enrichments). Still, mineral water was used to clean the palate between samples.

### 2.4. Statistical Analysis

Statistical analysis was performed using SPSS (SPSS 23.0, Chicago, IL, USA) software. The difference between mean values was tested using one-way ANOVA and Tukey’s post hoc test (*p* < 0.05).

## 3. Results and Discussion

### 3.1. Chemical Composition of CSC

The proximate composition of the control CSC and samples made with different algae are given in Table 2. Although micro- and macroalgae contain a high percentage of protein, this was not reflected in any of the batches with different types of algae. Despite a slightly elevated protein content in samples with algae, the observed difference was not statistically significant (*p* > 0.05). According to Serbian legislation (Serbian Regulations 34/2023) [30], which set a minimum protein content of 14% for cevaps, none of the formulations contained values that differed from this regulation. The addition of the same alga types to meat products in other studies showed similar results. The protein levels in salt-reduced frankfurters [31] and meat emulsions [32] were unaffected by the inclusion of wakame at 2% and 5%, respectively. Similarly, the protein content was not impacted by the addition of sea spaghetti in meat emulsion systems at 5% [32], pork sausages at 5% [33], and poultry steaks at 3% [34]. However, the protein content of the frankfurters was enhanced by almost 1% after the addition of 3% of white and honey *Chlorella* [16], while an even greater increase was found in beef burgers when the same algae was added [35].

Given the naturally low-fat content of algae, their inclusion was anticipated to have a negligible effect on the overall fat content of the products. The same was concluded in other studies, where the inclusion of sea spaghetti in poultry steaks (3%) [34], pork sausages (5%) [33], and salt-reduced frankfurters [17] did not have a significant impact on fat levels. Similarly, the incorporation of wakame showed no significant difference after being added to salt-reduced frankfurters (2%) [31] and meat emulsion systems (5%) [18]. Conversely, adding low-fat algae decreased the lipid content in the products. Both white and honey *Chlorella* (3%) demonstrated the ability to reduce fat content by more than 1% in frankfurters [16].

The moisture content decreased significantly (*p* < 0.05) in the samples with 3% of sea spaghetti (S3) and wakame (W3), compared to the control, while the highest results were observed in the control samples since algae have high dietary fiber content, which can lower the moisture of the meat product. Adding the same types of seaweed to frankfurters (1%) [36] and pork sausages (2.5%) [33] resulted in similar findings. In our study, *Chlorella* lowered the moisture content insignificantly, on the other hand, Bošković Cabrol et al. [16] found that this microalgae (3%) decreased moisture by nearly 1% in frankfurters.

The salt content ranged from 2.57 g/100 g in the control sample, to 3.09 g/100 g in W3. Being rich in NaCl, the inclusion of all three algae led to elevated salt levels in each CSC, despite a reduction of the added salt content in the recipe. This suggests that there is still potential for further decreasing the overall salt content in the recipe.

### 3.2. pH, a_w_, and Cooking Loss

The pH values were monitored over seven days under refrigerated conditions, with similar values observed across all samples at the start; however, differences became apparent as storage time progressed. By the seventh day, all the batches showed significantly different values (*p* < 0.05); samples containing *Chlorella vulgaris* exhibited the lowest pH values among those with added algae, while samples with wakame showed pH values closest to the control (Table 3). The direction of this effect is not consistently predictable, as researches has shown different trends. The incorporation of 2% Wakame into salt-reduced frankfurters resulted in a modest but statistically significant pH increase of 0.10 [31]. Similarly, Cofrades et al. [32] observed a slightly smaller rise in pH when a 2.5% concentration of the same macroalgae was added to low-salt gel/emulsion meat systems. In contrast, a 2.5% addition of sea spaghetti did not significantly affect pH values, whereas Porphyra umbilicalis led to a notable decrease in pH. The use of Laminaria japonica powder at concentrations of 5% in patties [37] and 4% in breakfast sausages [38] caused a reduction in pH by 0.10. Meanwhile, in pork liver pâtés, the addition of 2.5% spirulina had no observable effect on pH levels [39].

In terms of a_w_, only samples with higher amounts of sea spaghetti and wakame showed significantly lower values (*p* < 0.05) compared to the control, due to the high dietary fiber content of these algae. The type and amount of polysaccharides in the dietary fiber fractions of algae influence their gelation properties [40]. These properties also contributed to reduced cooking loss, particularly in samples with sea spaghetti and wakame, where the reduction was proportional to the concentration of added seaweed. On the other hand, *Chlorella* did not show statistically significant changes (*p* > 0.05) regardless of the concentration in the product. In earlier studies, a decrease in cooking loss was observed in meat patties [37] and breakfast sausages [38] with the addition of 3% *Laminaria japonica*. Similarly, the incorporation of three types of macroalgae (*Kappaphycus alvarezii*, *Sargassum polycystum*, and *Caulerpa lentillifera*) into chicken sausages led to a reduction in cooking loss, which was directly proportional to the algae concentration used (2%, 4%, and 6%) [41].

### 3.3. Texture

Regarding textural parameters, the hardness and chewiness of the samples with algae showed great differences, compared to control samples (Table 4). Batches prepared with *C. vulgaris,* and sea spaghetti resulted in a progressive decrease of hardness and chewiness, whereas the samples containing wakame were notably harder and chewier (*p* < 0.05) compared to the control. The data suggest that the increased hardness and chewiness may be correlated with the dietary fiber content in wakame, which is estimated to be around 34%, as reported by López-Santamaría et al. [42], while the other two types of algae have around three times lower dietary fiber content [13]. Furthermore, cohesiveness was slightly reduced (*p* < 0.05) in C and S, while W showed no significant difference. Changes in terms of springiness were observed only in C.

Incorporating white *Chlorella* (3%) into frankfurters did not have a significant impact on hardness and chewiness at the beginning of cold storage; however, these textural parameters increased by the end of the 60-day storage period. Notably, a significant decrease was demonstrated in cohesiveness and springiness [16]. In the case of grilled minced meat products, pork patties showed significantly higher values for hardness, springiness, and chewiness, after the addition of 3% *Laminaria japonica* [37], while the breakfast sausages observed similar results [38]. Contrary to our findings, sea spaghetti increased the hardness and chewiness of pork sausages [33], while the addition of wakame also resulted in greater hardness and chewiness in the meat batters [32], similar to our study.

As indicated by de Medeiros et al., the effect of incorporating algae varies with the type of meat product [43]. Furthermore, factors such as the type and concentration of the algae, the matrix of the meat products, and the thermal processes applied (e.g., drying, cooking, grilling, etc.) play significant roles in determining the functionality of algae in various meat products, ultimately affecting their textural properties.

### 3.4. Color

Meat color is a critical factor in consumers’ perceptions of quality, and although it cannot be regarded as a reliable indicator of overall quality, it significantly affects purchasing decisions [44]. The color parameters of the CSC (surface and cross-section) are presented in Table 5.

The surface lightness (L*) values of the samples containing algae were significantly lower (*p* < 0.05) than in the control, with the lowest value observed in sample W3. A notable decrease in surface redness (a*) and yellowness (b*) was detected in W1.5 and W3, while S3 exhibited a significantly lower a* value compared to the control (*p* < 0.05). The total color difference was the highest in W3 and the lowest in S3. A similar trend was noted for the cross-section values, where W3 again had the lowest L* and a* values, while samples with *C. vulgaris* remained unchanged. In contrast, only S1.5 and S3 had significantly higher b* values (*p* < 0.05). For the cross-section, ΔE was again greatest for W3, but the lowest value was recorded for C1.5.

The biggest influence on the color of the samples was attributed to the addition of wakame, likely due to the high concentration of pigments present and the darker natural color of this macroalgae compared to the other varieties used in this study. A similar observation was reported in another study, where the incorporation of just 1% wakame resulted in a higher ΔE, as all three color components significantly decreased in reformulated frankfurters [36], while in a cooked pork gel/emulsion system, 2.5% of this macroalgae decreased the lightness and redness, but increased the yellowness [32]. The same trend was found in frankfurters after incorporating 3% of white *C. vulgaris* into frankfurters, although the color difference was very moderate since chlorophyll-deficient microalgal biomass was added [16]. In a recent study, pork sausages with 2.5% sea spaghetti had lower scores for L*, a*, and b* values [33], a pattern also evident in our samples when we added a higher concentration of this algae.

### 3.5. Sensory Evaluation

In recent years, increased health awareness and promotion have significantly influenced consumer behavior and nutrition trends. This shift in purchasing intentions has prompted the meat industry to reformulate products to enhance their health benefits. On the other hand, the introduction of non-traditional ingredients has unfavorably affected the sensory quality of these reformulated products [45,46], as the ingredients in traditional formulations are crucial for developing the sensory characteristics that consumers prefer.

**Figure 1:**
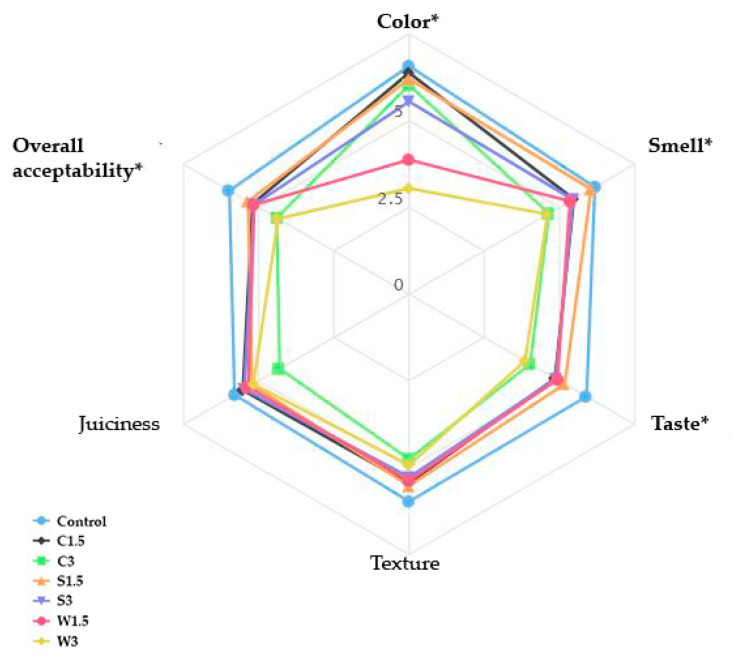
Results of sensory evaluation. C1.5—CSC with 1.5% *C. vulgaris*, C3—CSC with 3% *C. vulgaris*, S1.5—CSC with 1.5% sea spaghetti, S3—CSC with 3% sea spaghetti, W1.5—CSC with 1.5% wakame, W3—CSC with 3% wakame; * indicates a significant difference *p* < 0.05.

Similar findings were obtained after the sensory analysis in the present study, in which different algae in two concentrations had a significant influence (*p* < 0.05) on four out of six evaluated parameters (Figure 1). Although there were no significant differences in texture and juiciness (*p* > 0.05), it is worth mentioning that C3 received the lowest scores, more than 1.0 lower compared to the control samples for both parameters. These results are in correlation with TPA since C3 also showed the lowest values for hardness, cohesiveness, and chewiness.

In terms of color, the results showed that samples with added wakame were the least likable compared to the rest of the CSCs, similar to what CVS analysis showed. The panelists noticed the unusually dark green color of the products, which was their biggest complaint before consumption. As expected, W1.5 had a slightly higher score (3.9), while W3 had under 3, which are both significantly lower (*p* < 0.05) compared to the control CSC with a score of 6.2. A similar trend was found when wakame was added to frankfurters in different concentrations, where the results showed lower color scores with an increasing share of the algae [31]. Samples with other types of algae were not unattractive to consumers based on color.

Concerning smell and taste, C3 and W3 were the least desirable due to their earthy and marine flavors, with scores lower than 1.5 compared to the CSC without algae, while other samples showed relatively high acceptance scores. The overall acceptability matches the results of the previous parameters, where samples with 3% *C. vulgaris* and 3% wakame both had scores around 4.5, which is significantly lower (*p* < 0.05) than the control (6.0). In previously published studies, the overall sensory acceptability significantly decreased in cooked sausages containing sea spaghetti at 5% [33,36], as well as in frankfurters containing 1% of wakame [36], but despite achieving lower scores, 3% of *C. vulgaris* in frankfurters were evaluated as sensorily acceptable [16].

The low scores for C3 and W3 (~4.5) indicate reduced consumer acceptability, likely limiting market success. Adjusting algae concentrations or using masking strategies could help improve sensory appeal and align with consumer preferences. Wakame’s dark green color was unappealing and highlighted as a major issue. Its strong marine smell and flavor further contributed to its lower sensory scores, making it less acceptable to panelists. On the other hand, the rest of the formulations received scores above 5, which indicates the possibility of algae addition to meat products, with a focus on the type and added concentration, without significantly reducing the sensory characteristics of the product.

## 4. Conclusions

The results indicated that while the addition of algae did not significantly enhance the crude protein content, certain formulations exhibited noticeable modifications in moisture levels, pH, cooking loss, texture, color, and consumer acceptability.

The incorporation of *C. vulgaris* and sea spaghetti led to reductions in hardness and chewiness, suggesting their potential for producing more tender products. In contrast, the presence of wakame resulted in higher hardness and chewiness, which may be attributed to its dietary fiber content, while this macroalgae significantly affected the visual appeal of the products.

The sensory evaluation highlighted that while formulations with lower concentrations of algae were generally well-accepted, higher concentrations, particularly of *C. vulgaris* and wakame, were associated with lower overall acceptability scores. This relationship highlights the essential need to balance health benefits with sensory qualities, as the incorporation of non-traditional ingredients can detrimentally impact consumer acceptance.

Based on the results of this research, we can conclude that wakame is the least suitable for incorporation into the CSC, regardless of its concentration in the product, while *Chlorella* and sea Spaghetti showed significantly smaller deviations compared to the control sample, especially in lower concentrations. Further research is necessary in samples with these algae for a more detailed analysis of nutritional parameters, such as fatty acid, amino acid, and mineral composition.

## Figures and Tables

**Table 1:** Formulation of CSC with different algae per batch.

Ingredients (g)	Groups						
	Control	C1.5	C3	S1.5	S3	W1.5	W3
Beef meat (R II)	2450	2450	2450	2450	2450	2450	2450
Pork meat (S III)	2000	2000	2000	2000	2000	2000	2000
Ice	550	550	550	550	550	550	550
Salt	80	78.5	75	78.5	75	78.5	75
Dextrose	27.5	27.5	27.5	27.5	27.5	27.5	27.5
White *C. vulgaris*	/	75	150	/	/	/	/
Sea spaghetti	/	/	/	75	150	/	/
Wakame	/	/	/	/	/	75	150

C1.5—CSC with 1.5% *C. vulgaris*, C3—CSC with 3% *C. vulgaris*, S1.5—CSC with 1.5% sea spaghetti, S3—CSC with 3% sea spaghetti, W1.5—CSC with 1.5% wakame, W3—CSC with 3%.

**Table 2:** Proximate composition of CSC.

Parameters	Control	C1.5	C3	S1.5	S3	W1.5	W3
Protein (%)	15.60 ± 0.40	15.67 ± 0.32	15.90 ± 0.10	15.70 ± 0.20	15.73 ± 0.25	15.87 ± 0.30	15.90 ± 0.35
Fat (%)	13.97 ± 0.29	13.93 ± 0.28	14.10 ± 0.32	14.08 ± 0.24	14.08 ± 0.26	14.02 ± 0.16	14.03 ± 0.17
Moisture (%)	67.72 ± 0.83 ^a^	67.36 ± 0.98 ^a^	66.72 ± 1.05 ^a^	67.27 ± 0.72 ^a^	64.74 ± 0.85 ^b^	66.90 ± 1.16 ^a^	65.00 ± 1.05 ^b^
NaCl (g/100 g)	2.57 ± 0.17 ^a^	2.73 ± 0.18 ^ab^	2.60 ± 0.15 ^a^	2.77 ± 0.10 ^ab^	2.87 ± 0.03 ^ab^	2.79 ± 0.06 ^ab^	3.09 ± 0.16 ^b^

C1.5—CSC with 1.5% *C. vulgaris*, C3—CSC with 3% *C. vulgaris*, S1.5—CSC with 1.5% sea spaghetti, S3—CSC with 3% sea spaghetti, W1.5—CSC with 1.5% wakame, W3—CSC with 3% wakame. Values (means ± standard deviation) in the same row with different superscripts are significantly different (*p* < 0.05).

**Table 3:** pH, a_w_, and cooking loss of CSC.

Parameters	Control	C1.5	C3	S1.5	S3	W1.5	W3
pH day 0	5.63 ^ab^	5.62 ^a^	5.62 ^a^	5.61 ^a^	5.60 ^a^	5.67 ^b^	5.68 ^b^
pH day 1	5.66 ^a^	5.65 ^ab^	5.64 ^ab^	5.64 ^ab^	5.62 ^bc^	5.70 ^cd^	5.72 ^d^
pH day 3	5.54 ^ab^	5.48 ^ab^	5.4 ^a^	5.52 ^ab^	5.58 ^abc^	5.60 ^bc^	5.67 ^c^
pH day 7	5.36 ^a^	5.00 ^bc^	4.96 ^c^	5.11 ^de^	5.08 ^cd^	5.19 ^ef^	5.20 ^f^
a_w_	0.983 ^a^	0.977 ^ab^	0.978 ^ab^	0.977 ^ab^	0.972 ^b^	0.977 ^ab^	0.970 ^b^
CL (%)	23.06 ^ab^	24.21 ^a^	23.81 ^a^	22.74 ^ab^	20.79 ^b^	17.15 ^c^	12.40 ^d^

C1.5—CSC with 1.5% *C. vulgaris*, C3—CSC with 3% *C. vulgaris*, S1.5—CSC with 1.5% sea spaghetti, S3—CSC with 3% sea spaghetti, W1.5—CSC with 1.5% wakame, W3—CSC with 3% wakame. Values (means ± standard deviation) in the same row with different superscripts are significantly different (*p* < 0.05).

**Table 4:** Texture profile analysis parameters of the CSC.

Parameters	Control	C1.5	C3	S1.5	S3	W1.5	W3
Hardness (N)	20.39 ± 1.98 ^a^	17.47 ± 1.87 ^b^	14.34 ± 1.82 ^c^	16.10 ± 1.74 ^bd^	15.79 ± 1.99 ^cd^	22.16 ± 1.99 ^e^	24.21 ± 2.03 ^f^
Cohesiveness	0.44 ± 0.03 ^a^	0.40 ± 0.04 ^b^	0.36 ± 0.03 ^c^	0.40 ± 0.03 ^b^	0.37 ± 0.04 ^bc^	0.46 ± 0.02 ^a^	0.46 ± 0.03 ^a^
Springiness (mm)	1.00 ± 0.01 ^a^	1.06 ± 0.08 ^b^	1.05 ± 0.07 ^b^	1.04 ± 0.06 ^ab^	1.03 ± 0.05 ^ab^	1.01 ± 0.02 ^a^	1.01 ± 0.02 ^a^
Chewiness (N × mm)	9.25 ± 0.99 ^a^	7.57 ± 1.00 ^b^	5.52 ± 0.74 ^c^	6.72 ± 0.84 ^d^	5.92 ± 1.02 ^c^	10.22 ± 1.09 ^e^	11.33 ± 1.21 ^f^

C1.5—CSC with 1.5% *C. vulgaris*, C3—CSC with 3% *C. vulgaris*, S1.5—CSC with 1.5% sea spaghetti, S3—CSC with 3% sea spaghetti, W1.5—CSC with 1.5% wakame, W3—CSC with 3% wakame. Values (means ± standard deviation) in the same row with different superscripts are significantly different (*p* < 0.05).

**Table 5:** Color parameters and color differences of the CSCs.

	Control	C1.5	C3	S1.5	S3	W1.5	W3
				Surface			
L*	34.67 ± 6.30 ^a^	29.37 ± 6.57 ^b^	31.47 ± 7.19 ^ab^	30.77 ± 6.50 ^ab^	28.63 ± 6.23 ^b^	27.50 ± 5.72 ^b^	16.93 ± 6.72 ^c^
a*	8.20 ± 2.32 ^ab^	8.20 ± 2.28 ^ab^	8.77 ± 1.75 ^a^	8.70 ± 2.07 ^a^	6.87 ± 1.38 ^bc^	6.00 ± 2.68 ^c^	2.07 ± 1.84 ^d^
b*	21.43 ± 3.58 ^a^	20.13 ± 3.10 ^ab^	21.83 ± 3.26 ^a^	22.37 ± 3.24 ^a^	19.70 ± 3.82 ^ab^	17.63 ± 3.81 ^b^	12.47 ± 3.59 ^c^
ΔE		9.42 ± 3.13 ^a^	10.11 ± 5.02 ^a^	9.83 ± 5.00 ^a^	8.39 ± 4.02 ^a^	11.28 ± 3.86 ^a^	21.49 ± 6.01 ^b^
				Cross			
L*	41.00 ± 5.14 ^a^	41.07 ± 4.51 ^a^	41.30 ± 4.53^a^	37.83 ± ^ab^	34.33 ± 6.18 ^bc^	32.50 ± 7.19 ^c^	24.23 ± 7.05 ^d^
a*	7.97 ± 1.92 ^ab^	8.53 ± 1.38 ^a^	6.97 ± 2.11 ^abc^	6.77 ± 2.34 ^bc^	5.97 ± 2.52 ^c^	2.77 ± 2.04 ^d^	0.67 ± 2.84 ^e^
b*	14.47 ± 2.21 ^a^	15.13 ± 1.72 ^ab^	15.90 ± 2.52 ^abc^	17.10 ± 2.71 ^bc^	17.90 ± 2.50 ^c^	15.57 ± 3.09 ^ab^	15.50 ± 3.63 ^ab^
ΔE		6.01 ± 2.65 ^a^	7.03 ± 2.85 ^ab^	7.68 ± 3.08 ^ab^	9.39 ± 3.96 ^b^	12.66 ± 4.88 ^c^	18.67 ± 5.44 ^d^

C1.5—CSC with 1.5% *C. vulgaris*, C3—CSC with 3% *C. vulgaris*, S1.5—CSC with 1.5% sea spaghetti, S3—CSC with 3% sea spaghetti, W1.5—CSC with 1.5% wakame, W3—CSC with 3% wakame. Values (means ± standard deviation) in the same row with different superscripts are significantly different (*p* < 0.05).

## Data Availability

The original contributions presented in the study are included in the article, further inquiries can be directed to the corresponding authors.
